# An Unusual Developmental Profile of Salla Disease in a Patient with the SallaFIN Mutation

**DOI:** 10.1155/2012/615721

**Published:** 2012-11-22

**Authors:** Liisa E. Paavola, Anne M. Remes, Pirkko H. Sonninen, Vesa V. Kiviniemi, Tapio T. Korhonen, Kari Majamaa

**Affiliations:** ^1^Department of Neurology, Oulu University Hospital, P.O. Box 20, 90029 Oulu, Finland; ^2^Department of Clinical Medicine, Neurology, University of Oulu, P.O. Box 5000, 90014 Oulu, Finland; ^3^Department of Neurology, Institute of Clinical Medicine, University of Eastern Finland, P.O. Box 1627, 70211 Kuopio, Finland; ^4^Department of Neurology, Kuopio University Hospital, P.O. Box 1777, 70211 Kuopio, Finland; ^5^Medical Imaging Centre, Turku University Hospital, P.O. Box 52, 20521 Turku, Finland; ^6^Department of Radiology, Oulu University Hospital, P.O. Box 20, 90029 Oulu, Finland; ^7^Division of Psychology, Department of Behavioural Sciences and Philosophy, University of Turku, 20014 Turku, Finland

## Abstract

Salla disease (SD) is a disorder caused by defective storage of free sialic acid and results from mutations in the *SLC17A5* gene. Early developmental delay of motor functions, and later cognitive skills, is typical. We describe a developmental profile of an unusual homozygous patient, who harboured the SallaFIN (p.R39C) mutation gene. The study involved neurological examination, neuropsychological investigation, and brain imaging. The neurocognitive findings were atypical in comparison with other patients with the SallaFIN mutation. Interestingly, there was no deterioration in the patient's neurological condition during adulthood. Her neurocognitive skills were remarkably higher than those of other patients with a conventional phenotype of SD. Our results suggest that the phenotype of SD is broad. Unidentified genetic or environmental variation might explain the unique SD type of this case.

## 1. Introduction

Salla disease (SD; OMIM 604369) is a disorder characterised by defective storage of free sialic acid and belongs to the Finnish disease heritage [1]. However, sporadic cases of SD have been reported in many countries. The disease is caused by mutation of the *SLC17A5* gene, which encodes a protein that transports sialic acid across the lysosomal membrane [2]. 

Salla disease affects the white matter by causing dysmyelination of the central nervous system and the peripheral nervous system. Magnetic resonance imaging studies have shown dysmyelination of the entire white matter of the cerebrum [3]. Cerebellar involvement has also been reported [4]. Hypoplasia of the corpus callosum is a typical finding in patients with SD. A conventional subtype and a severe subtype of the disease have been identified [5]. 

In neurocognitive terms, SD impacts nonverbal performance more than linguistic ability [6]. The common features related to nonverbal learning disabilities are associated with dysmyelination of the white matter. Another typical pattern seen in patients with SD is severe motor disability. After the second decade of life, the decline in motor skills is usually more pronounced than that in cognitive function. All affected individuals are intellectually disabled, but the level of cognitive and motor disabilities varies notably among patients with SD. 

It is estimated that 95% of Finnish patients with SD are homozygous for the SallaFIN mutation: the p.R39C allele of the *SLC17A5* gene [7]. Only a few patients are compound heterozygotes. These patients harbour the SallaFIN mutation in one allele of *SLC17A5* and a different mutation in the other. Compound heterozygotes have a more severe phenotype than homozygotes [5]. Here we describe a patient with the homozygous SallaFIN mutation. Her neurocognitive development is unusual when compared with that of other patients with the same phenotype. 

## 2. Case Presentation

The proband is a 30-year-old woman. She was born after an uneventful pregnancy at full term. Her parents were nonconsanguineous. SD was diagnosed at 3 years of age on the basis of clinical symptoms and increased level of free sialic acid in the urine.

The patient's development during the first year of life was relatively normal, but crawling was unstable and muscular hypotonia and nystagmus were noticed. The patient spoke her first words at 1 year of age and her first sentences at 2 years of age. She learned to walk by 1.5 years of age, but her gait and balance were abnormal. At 3 years of age, her cognitive development was assessed as normal, except for mild slowness and clumsiness when performing fine motor skills. The followup evaluations showed mild delays in motor tasks, eye-hand coordination, and concentration. Her verbal development was slightly delayed, and verbal dyspraxia was reported. At 6 years of age, the developmental delay was approximately 2 years.

Inattentiveness, hyperactivity, and problems with sleep were reported during childhood. The patient also had problems with balance and body awareness. Ataxic symptoms were prominent in childhood, but improved during the teenage years. 

During her school years, the neurocognitive development fluctuated notably. Verbal performance was consistently better than visual performance or fine motor skills. Intellectual disability was considered to be mild. 

At the age of 12 years, the patient's verbal skills, as assessed using the Wechsler Intelligence Scale for Children-R test [8], were at the level of a 7 year old, and her performance skills varied between those typical of a child of 5 years 6 months and 6 years 6 months of age. Two years later, her verbal skills had improved. At 14 years of age, no progression was noted in the neurocognitive deficits. The developmental age of the patient varied between 4 and 8 years, and her verbal skills were notably better than her motor and visual abilities. 

### 2.1. Neurological Examination

At the age of 30 years, the proband was living alone with support. She was a social person, keen on the arts and team sports. She was 157 cm in height and weighed 56 kg. She was taking no medications. On examination, auscultation of the heart and lungs was unremarkable, her blood pressure was 114/74 mmHg, and the electrocardiogram was normal. Her facial features were slightly coarse. The proband could walk without aid, but both legs were in a pes planus position. When walking, she had some athetotic movements in her upper extremities. Muscle strength and skin sensation were normal, tendon reflexes were symmetrical and normal, and the plantar responses were in flexion. Both Achilles tendons were slightly shortened and there was mild spasticity in both legs. Neurological examination revealed only mild ataxia. There was mild instability in the Romberg test and the patient was unable to stand with her eyes closed. There was no ataxia or dysmetria shown by coordination tests, but her hand movements were clumsy. She suffered from marked myopia and used six dioptre corrective lenses. Clear outward strabismus was seen in her right eye. However, the eye movements were normal and nystagmus was not detected. The neurological condition of the patient had not deteriorated during the previous 10 years. 

There had been no deterioration of the patient's motor skills in adulthood. Her skills had improved with respect to balance, coordination of body movements, and reciprocal motor actions, as well as processing the sequences of movements. The speed of motor actions had become slightly slower during the last few years.

The electroencephalogram (EEG) was normal at 3 years of age, but showed mild generalized background abnormality with occasional spikes and sharp waves at the left temporo-parieto-central region at 5 years of age. Quantitative EEG was normal at 15 years of age. There was no history of epileptic seizures, but symptoms that resembled the startle reflex were noticed in response to sudden noises.

### 2.2. Neurocognitive and Motor Development

The methods that were used for neuropsychological evaluation of the patient are presented in Table 1. Her developmental age, as assessed by Wechsler Intelligence Scale for Children-III [9] at the age of 30 years, was 7 years 9 months for the verbal scale and 5 years 4 months for the perceptual scale. Her neurocognitive performance was remarkably better than those of other patients with the conventional type of SD and the SallaFIN mutation [6]. Other patients with SD (*n* = 37) have been evaluated using the Bayley Scales of Infant Development-II [14], because the tasks that are used in the Wechsler children's tests were too demanding. 

There was a slowing in visuomotor speed as well as eye-hand coordination during the followup of our patient after her teenage years. Visual reasoning and spatial orientation were mildly delayed, and the visuoconstructive skills were diminished. However, verbal skills had improved. Repetition of nonsense words and oromotor sequences were difficult for the proband because of verbal and oral dyspraxia, but the proband was able to learn and repeat long, logical stories. She had difficulties with time orientation. 

Motor problems were evident but the symptoms had not progressed during the followup. The proband was able to walk on the toes and sides of the feet, but the forward tandem walk was insecure, and motor persistence and motor coordination were clumsy. Static cerebellar tests were performed quite well, with only slight problems with balance. Two of the dynamic cerebellar tests—finger-to-thumb tapping and toe tapping—were performed slowly but correctly. Visuomotor deficits were evident, but the proband managed the test of basic functional mobility quite well. 

### 2.3. Brain Imaging

**Figure 1:**
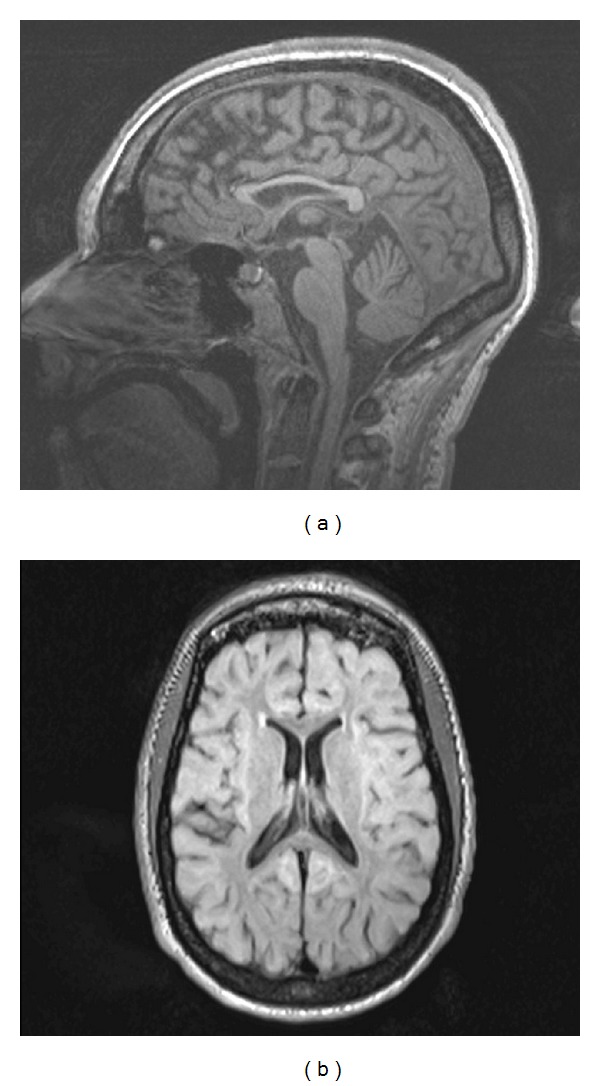
Hypoplastic corpus callosum in magnetic resonance imaging of the patient. (a) sagittal plane; (b) axial plane.

Brain imaging performed at 15 years of age showed dysmyelination. The corpus callosum was hypoplastic, but the cerebellum, pons, and the proximal part of the cervical cord were normal. There was no enlargement of the ventricles or signs of cortical atrophy. The MRI findings at 30 years of age were mild (Figure 1). 

## 3. Discussion

The typical neurocognitive profile of SD consists of a lower level of nonverbal performance as compared with linguistic skills. Findings related to nonverbal learning disabilities have been outlined [6]. Herein, we have described an unusual developmental profile of SD in a patient with the SallaFIN mutation. Her neurocognitive development differs from that of other patients with SD of the conventional subtype who carry the SallaFIN mutation. Her neurological condition has remained fairly constant during adulthood. Only mild progression of the symptoms related to her neurocognitive skills has been seen. The MRI findings showed that the ventricles were of normal size; the corpus callosum was thin, but there was no cortical atrophy. 

The patient has received regular physiotherapy and has participated actively in sports since childhood. The benefits to the brain of physical activity, which include anatomical, functional, and molecular changes, have been documented [15]. Physical activity also affects the health of the neural network and on the capacity to process information. 

Unknown genetic and environmental variation might explain the unique SD type of the proband. The heterogeneity of the severity and progression of SD is a challenge for diagnostic work and rehabilitation with both children and adult patients.

## Figures and Tables

**Table 1:** Neuropsychological evaluation of the proband at 30 years of age.

Full name of the test	Abbreviation	Reference	The domain of the test/chosen parts	Results^a^
Wechsler Intelligence Scale for Children-III	WISC-III	Wechsler (1991) [9]	*Verbal skills	1
			*Perceptual skills	1
Children's Neuropsychological Test Battery	NEPSY	Korkman et al. (1997) [10]	*Comprehension of instructions	0
			*Oromotor sequences	2
			*Repetition of nonsense words	2
Physical and Neurological Examination of Soft Signs	PANESS	Denckla (1985) [11]	Corpus callosum dysfunction	2
Static and Dynamic Cerebellar Tests	Cerebellar tests	Fawcett et al. (2001) [12]	Dysfunction of cerebellum	1
Timed Up-and-Go-test	TUG-test	Williams et al. (2005) [13]	Basic and functional mobility	1

^a^0 = among average, 1 = mild deficits, 2 = severe deficits.
